# Unsupervised machine learning for cardiovascular disease: A framework for future studies

**DOI:** 10.1002/ejhf.70076

**Published:** 2025-11-06

**Authors:** Emmanuel Bresso, Claire Lacomblez, Kévin Duarte, Luca Monzo, Guillaume Baudry, Jasper Tromp, Abhinav Sharma, Nicolas Girerd

**Affiliations:** ^1^ Université de Lorraine, INSERM, Centre d'Investigations Cliniques Plurithématique 1433, Inserm U1116, CHRU de Nancy and F‐CRIN INI‐CRCT Nancy France; ^2^ Saw Swee Hock School of Public Health, National University of Singapore & National University Health System Singapore Singapore; ^3^ Division of Cardiology McGill University Health Centre, McGill University Montreal QC Canada

**Keywords:** Unsupervised machine learning, Clustering algorithms, Cardiovascular diseases, Patient stratification, Precision medicine

## Abstract

Unsupervised machine learning can improve the characterization and stratification of patients with cardiovascular diseases (CVDs). Clustering algorithms, which group patients based on patterns in clinical data, can reveal distinct subgroups that may differ in prognosis and treatment response. Despite increasing research in this area, the practical use of clustering methods in routine clinical care remains limited by the lack of accessible tools and rigorous external validation. This review presents a systematic framework for applying unsupervised machine learning techniques to CVD research. The framework outlines a stepwise process—from identifying patient clusters and establishing their associations with clinical outcomes to developing predictive models for assigning new patients to these clusters. This approach aims to generate robust, externally validated models that can be integrated into clinical practice to support improved risk stratification and personalized treatment strategies. This framework can enhance the usefulness of clustering in CVD research, by providing valuable resource for medical professionals, stakeholders, and researchers in exploring more effective strategies for managing CVDs.

## Introduction

Cardiovascular diseases (CVDs) continue to be a significant cause of mortality and morbidity worldwide, posing a substantial public health challenge.^1^ Early detection and accurate prediction of CVDs are essential for timely interventions and improved patient outcomes. In recent years, machine learning (ML) algorithms have been studied for their potential to enhance CVD risk assessment and prediction.^2^, ^3^

Machine learning approaches have emerged as powerful tools to enhance cardiovascular risk assessment, leveraging high‐dimensional data and complex interactions among clinical variables. Comprehensive reviews of the literature have been recently conducted on the subject of the benefits ML methods in cardiovascular prediction by Quer *et al*.,^4^ and Wang *et al*.^5^

Machine learning techniques can customize predictions for individual patients, analysing large and heterogeneous datasets, identifying patterns, and incorporating a broad spectrum of features to predict CVDs. The primary advantage of ML models is their ability to integrate non‐linear associations into prediction models, effectively capturing complex interactions and hidden risk factors.^6^, ^7^, ^8^ Unsupervised clustering, in particular, can separate patient subgroups with similar characteristics and outcomes within diverse demographics.^9^ Such methods are being applied to explore heterogeneous groups—for example, individuals with heart failure (HF) with preserved ejection fraction,^10^ coronary artery disease, acute HF^11^, ^12^, ^13^ or community‐based cohorts of otherwise healthy subjects.^14^

Machine learning‐based methods can identify subgroups that benefit differently from particular interventions, which could refine clinical trial design and interpretation. For example, clustering has been used to demonstrate treatment benefits in trials such as EPHESUS^15^ or scenarios with fewer expected events.^16^, ^17^ However, despite the increasing number of studies employing ML‐derived clusters,^18^ a critical limitation remains. Many models lack the power to predict clusters in populations beyond the derivation cohort. This limitation is a significant barrier to ensuring the findings are generalizable across different patient populations and healthcare settings. Additionally, most studies applying ML‐derived clusters have not conducted external validation nor tested prediction algorithms for cluster membership.^19^

**Figure 1:**
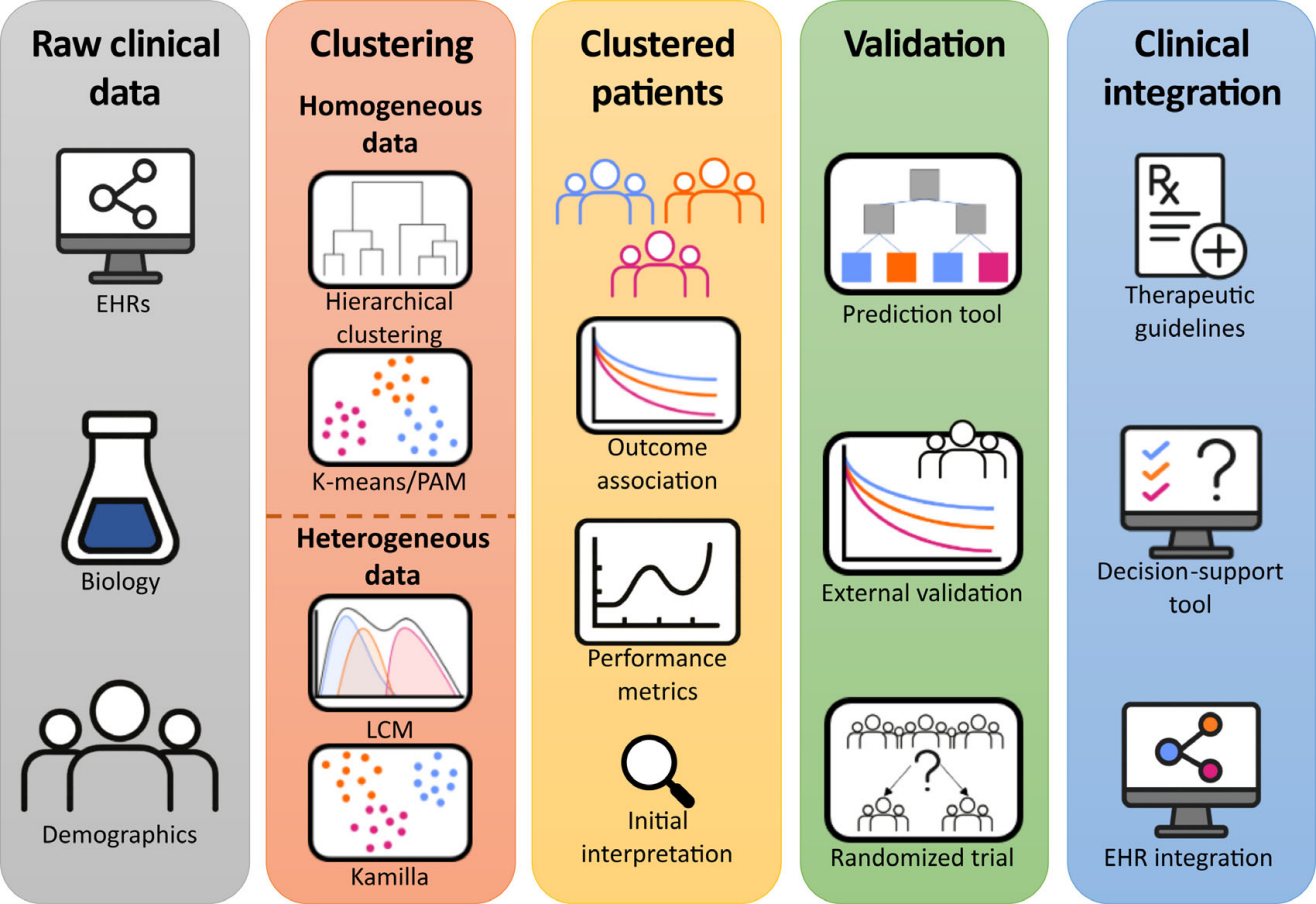
End‐to‐end workflow for unsupervised patient stratification in cardiovascular medicine. EHR, electronic health record; LCM, latent class model; PAM, partition around medoids.

Implementing clustering outcomes in everyday clinical settings remains challenging. Model robustness and reproducibility are often insufficient, and clinicians may not have the appropriate tools to apply the findings directly to their patients. To address these issues and account for the complexity of medical data, we propose a detailed framework using clustering methods in CVD research. *Figure* *1* summarizes the end‐to‐end workflow—from raw data and algorithm selection to validation and clinical integration. This framework is designed to identify patient subgroups that differ in prognosis or treatment response, thereby supporting improved management strategies and patient outcomes.

## Why clustering is a valuable tool in cardiovascular research: promises and pitfalls

While the rationale for employing clustering approaches may not always be explicitly stated in some studies, these techniques offer valuable insights in various clinical research contexts. Clustering provides a novel, data‐driven perspective on complex problems in cardiovascular research, ultimately aiming to improve patient care and advance our understanding of CVD. Below, we outline key objectives effectively addressed by clustering methods and associated pitfalls that can hinder their clinical utility (*Table* *1*).

**Table 1:** Objectives, promises, and pitfalls of clustering approaches in cardiovascular research

Objective	Promise	Pitfalls
Identifying specific disease profiles	Uncovers clinically meaningful subgroups, refining disease understanding and management	Poor data quality or inappropriate clustering methods can yield unreliable or biased clusters.
Improving risk stratification	Enhances predictive accuracy and guides targeted interventions	Lack of robust validation can result in inaccurate predictions and limited generalizability
Guiding therapeutic decisions	Enables precision medicine by identifying subgroups responsive to specific treatments	Insufficient external validation or practical barriers may limit clinical applicability.
Facilitating patient management	Provides dynamic, data‐driven support for clinical decisions through EHR integration	Operational complexity and interpretability issues can hinder routine clinical adoption.
Identifying novel therapeutic targets	Reveals new biological insights and therapeutic avenues	Clusters derived from limited diversity or inadequate validation may lead to misleading or unproductive insights.

EHR, electronic health record.

### Identifying specific disease profiles

Disease classification in medicine is traditionally based on predefined syndromes and diagnostic categories, often influenced by historical precedent, expert opinion, or arbitrary thresholds rather than a purely systematic, data‐driven approach. In HF, for example, the primary classification relies on left ventricular ejection fraction. However, such classifications may overlook nuanced subgroups with distinct pathophysiological features. This is a recurring debate in various diseases such as HF with preserved ejection fraction and chronic coronary disease.

Clustering provides a systematic opportunity to identify hidden patient profiles that conventional classification methods might have previously missed. These clustering‐derived profiles play two critical roles. First, they validate existing segmentation methods by confirming their alignment with underlying pathophysiology. Second, they offer operational benefits by identifying subgroups that may benefit from personalized interventions, thus promoting more effective and targeted patient management. Nonetheless, poor data quality or inappropriate clustering methods can hinder the identification of reliable, meaningful clusters, diminishing the overall utility of the findings.

### Improving risk stratification

Clustering can reveal subgroups of patients who share distinct risk profiles that may not be apparent through conventional scoring systems. By assigning new patients to these data‐driven clusters, clinicians can integrate cluster membership with traditional clinical parameters—such as ejection fraction and biomarker levels—to refine prognostication. This integrated approach provides a clearer view of patients at higher risk for clinical events, guiding more vigilant monitoring.^20^ Additionally, identifying clusters predisposed to early disease onset or rapid progression can prompt more targeted and timely interventions.^14^ However, inadequate validation may result in clusters with limited predictive power and generalizability, potentially compromising their practical clinical utility.

### Guiding therapeutic decisions in precision medicine

Clinicians can leverage this information to personalize therapies based on cluster membership when patient clusters exhibit distinct treatment responses. Unlike traditional approaches focusing on single‐variable interactions, clustering integrates multiple variables simultaneously, offering a more comprehensive and biologically relevant way to identify subgroups with unique treatment responses. Because these clusters often capture underlying biological differences more effectively than isolated clinical markers, they can reveal novel treatment‐responsive subgroups that might otherwise go unnoticed. Moving beyond conventional, one‐variable‐at‐a‐time analyses, clustering provides a data‐driven framework for refining therapeutic choices and improving patient outcomes.^19^

If an algorithm identifies patient subgroups with distinct treatment responses not predicted by other clinical variables, its practical relevance becomes even more significant. ML tools capable of predicting treatment responses remain scarce. While some studies have explored similar analyses, they often do not share their developed tools, which limits broader application and validation.^21^ Ensuring the dissemination of these predictive tools is crucial for maximizing their utility and advancing precision medicine.

By comparing treatment outcomes, such as placebo versus active interventions, across distinct clusters, researchers can pinpoint which subgroups experience the most significant therapeutic benefit or, conversely, face a higher risk of harm. These insights can help optimize clinical trial design by enabling more targeted patient selection. A refined selection process reduces the sample sizes needed to observe meaningful effects while clarifying the magnitude of therapeutic benefit. Additionally, clustering enhances clinical monitoring and decision‐making by identifying patient subgroups that exhibit lower‐than‐expected responsiveness to a given treatment. In such cases, alternative therapeutic strategies or closer follow‐up may be necessary.

Several studies have applied cluster‐based analyses in clinical trials to explore these distinct responses.^22^, ^23^, ^24^ By systematically investigating cluster‐specific therapeutic effects, researchers improve a model's predictive accuracy and establish its potential to drive meaningful improvements in patient care. Yet, insufficient external validation or real‐world implementation barriers can limit the clinical impact of these clusters.

However, it is crucial to underscore that while these observational findings are promising, well‐designed randomized controlled trials (RCTs) specifically testing cluster‐guided treatment strategies remain essential before clinical implementation. The identification of treatment‐responsive subgroups through clustering represents only the hypothesis‐generation phase of precision medicine. These findings must be prospectively validated in appropriately powered RCTs where patients are randomized to cluster‐guided versus standard care approaches, with clinical outcomes as primary endpoints.

### Facilitating personalized patient management

When clustering proves valuable for both risk stratification and treatment selection, it can be seamlessly integrated into clinical decision‐making through electronic health records (EHR) and decision support systems. As patients transition between clinical states, such as following hospitalization, their cluster membership may change. An adaptive, auto‐updating model can alert clinicians to evolving risk profiles or emerging treatment opportunities, ensuring timely interventions. Additionally, by leveraging cluster characteristics, clinicians can provide patients with more accurate, data‐driven insights into their likely disease trajectory. This enhanced understanding fosters shared decision‐making, enabling patients to make more informed choices about interventions and lifestyle adjustments in collaboration with their healthcare providers. Despite these benefits, operational complexity and interpretability challenges may hinder the practical integration of clustering into routine clinical practice.

### Identifying novel pathways for targeted therapies

Clustering can also uncover unexpected clinical patterns or disease trajectories, opening new avenues for research and therapeutic innovation by biological target identification and mechanistic insights. If high‐risk clusters exhibit specific molecular, genetic, or biomarker profiles, these could serve as potential therapeutic targets. Researchers can further explore genetic variants, molecular pathways, or environmental exposures within clusters to gain deeper pathophysiological understanding, potentially leading to innovative treatment strategies. However, clusters derived from datasets lacking diversity or robust validation may yield misleading or unproductive biological insights, limiting the scientific and clinical value of clustering‐derived discoveries.

## A clustering framework to provide useable and useful results/tools from clustering approaches

**Figure 2:**
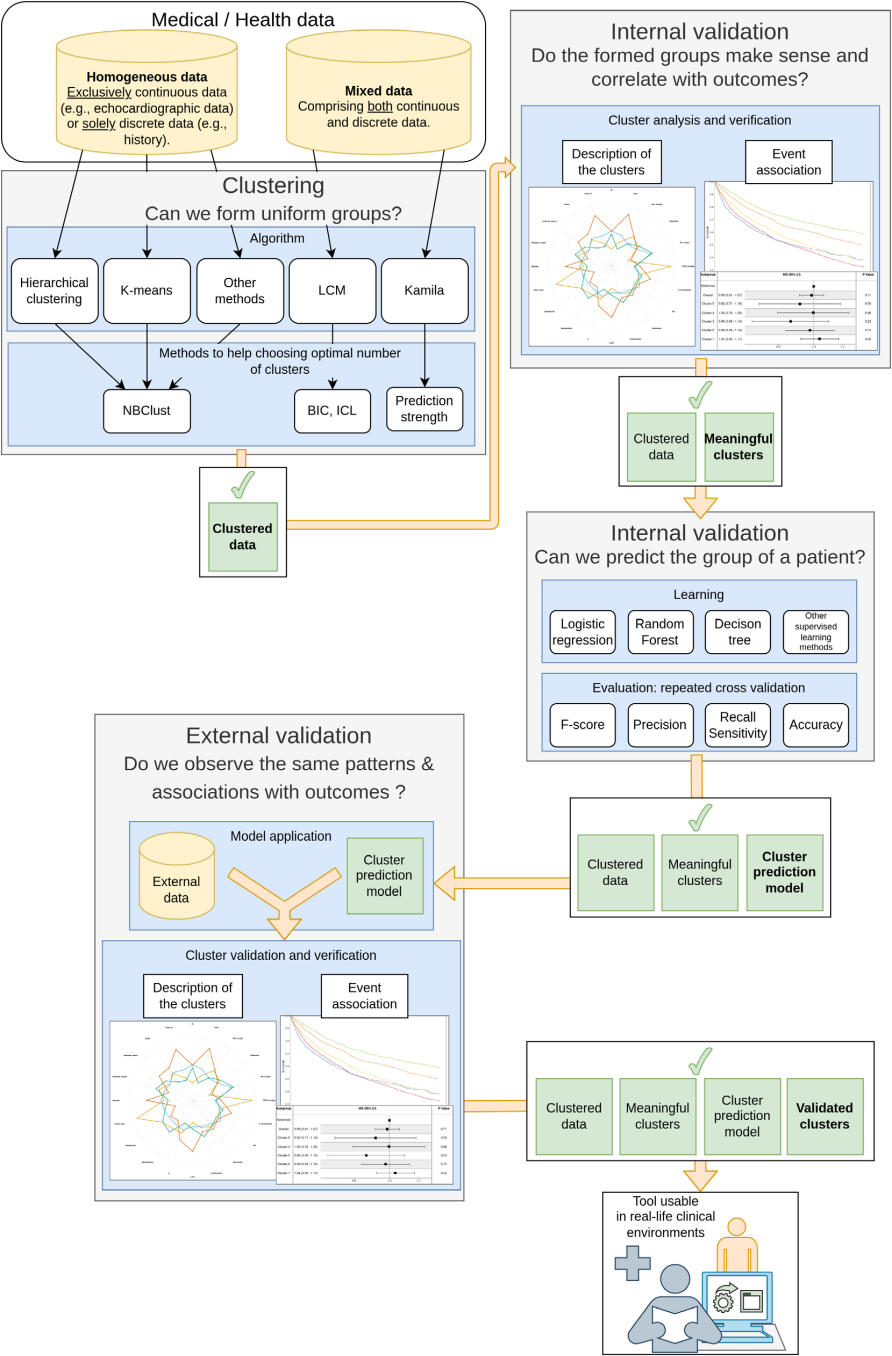
Methodological framework for clustering analysis and its clinical integration. BIC, Bayesian information criterion; ICL, integrated completed likelihood; LCM, latent class model.

The proposed framework, illustrated in *Figure* *2*, is centred on precisely identifying, validating, and utilizing clinically pertinent patient clusters. These clusters are methodically correlated with clinical outcomes to develop robust prediction models. These models are meticulously validated internally and externally to ensure their reliability and practical applicability in real‐world clinical settings.^25^

This framework employs state‐of‐the‐art clustering techniques that have been explicitly tailored for CVD research. Its objective is to translate complex clinical data into actionable patient insights with the potential to significantly enhance the implementation of personalized medicine. Supported by comprehensive R notebooks, the framework aims to promote widespread adoption among cardiovascular researchers by providing practical tools and step‐by‐step guides for implementing these techniques effectively.

A critical aspect of the framework is the rigorous validation process, which includes internal and external validations to ensure the robustness and generalizability of the findings. This process confirms the significant clinical relevance of the identified clusters, facilitating the development of predictive models that can accurately categorize patients into these clusters based on their unique clinical profiles. The successful application of this clustering framework has the potential to impact cardiovascular medicine profoundly. By offering a more profound comprehension of CVD subtypes and empowering refined risk stratification and prediction of treatment responses, this framework functions as a scientific and practical guide for researchers, healthcare providers, and stakeholders, paving the way for more effective and customized strategies for managing CVDs, which could ultimately improve patient outcomes.

An exemplary implementation of this framework using R is accessible at https://gitlab.com/cic‐p/cv‐clustering. This resource aims to facilitate the practical application of advanced clustering methods in cardiovascular research, providing researchers and practitioners with the tools to integrate these insights seamlessly into clinical practice.

## Data collection

### Data size determination

The effectiveness of clustering depends on having a sufficiently large sample to reveal statistically significant patterns. Dalmaijer *et al*.^26^ recommend a minimum of 20 to 30 patients per expected subgroup to ensure meaningful separation of clusters. This guideline is based on power analyses that consider the inherent variability of the data and the desired confidence in detecting differences between groups. Adhering to these recommendations facilitates the reliable identification of distinct patient clusters, which is crucial for subsequent predictive modelling and clinical applications.

In cases where there is significant overlap or heterogeneity among patients, a larger sample size is advisable—often exceeding 1000 patients. Large registries with over 10 000 patients are ideally suited to provide reliable clustering outcomes. Additionally, the number of variables included in the analysis is critical. Using too few variables may hinder precise patient cluster identification, while incorporating too many variables can lead to issues such as the ‘curse of dimensionality’ and impede the formation of homogeneous groups.^27^, ^28^ Therefore, variable selection must be carefully performed based on relevance to disease characteristics and predictive value.^29^ Techniques such as principal component analysis for dimensionality reduction, mutual information scores to evaluate feature importance, and consultation with domain experts are essential.^30^, ^31^ This careful selection process helps maintain model accuracy and computational efficiency, ensuring the identification of clinically meaningful patient clusters.

### Data management strategies

Effective data management is crucial for ensuring the integrity and reliability of clustering outcomes. If left unaddressed, missing data can bias outcomes; therefore, imputation strategies—ranging from simple mean or median replacement to more sophisticated approaches like regression‐based imputation or multiple imputation—help maintain consistent data patterns. Outliers, which can artificially skew cluster boundaries or inflate within‐cluster variability, are typically identified using both statistical (e.g. Z‐scores, interquartile range) and graphical (e.g. boxplots) techniques; once detected, these data points can be capped, transformed, or removed based on clinical plausibility. Finally, normalizing variables through min‐max scaling, Z‐score standardization, or logarithmic transformation prevents attributes with large numeric ranges from overshadowing those measured on smaller scales. A systematic application of these methods enhances clustering outcomes' robustness, interpretability, and overall reliability.

## Clustering tools

The first phase of the framework involves clustering cardiovascular data using methods that best match the data's characteristics. The choice of clustering algorithm and the determination of the optimal number of clusters are interdependent decisions central to practical unsupervised analysis in clinical and biomedical research. These decisions are largely dictated by the structure of the data, whether homogeneous (composed primarily of continuous variables) or heterogeneous (a mix of continuous and categorical variables) (*Table* *2*).^32^, ^33^, ^34^, ^35^, ^36^, ^37^, ^38^, ^39^

**Table 2:** Clustering methods and evaluation criteria for different data types

Data type	Algorithm	Description	Evaluation metrics for cluster number
Homogeneous	Hierarchical agglomerative clustering	Constructs a dendrogram to hierarchically represent clusters	Silhouette Score^32^ Calinski–Harabasz Index^33^ Davies–Bouldin Index^34^ NbClust Package^35^
	K‐means	Partitions data into *k* clusters by minimizing within‐cluster variance	
	Partition around medoids	Similar to k‐means but uses medoids, reducing sensitivity to outliers	
	Fuzzy C‐means	Allows data points to belong to multiple clusters	
Heterogeneous	Latent class model	Model‐based probabilistic approach. Identifies patient clusters using mixed data	Bayesian information criterion (BIC) Integrated completed likelihood (ICL)^36^, ^37^, ^38^
	Kamila	Modified k‐means for mixed data types	Prediction strength (cluster stability across subsamples)^39^

For homogeneous datasets, commonly encountered in physiological measurements or laboratory values, widely used algorithms include hierarchical clustering, K‐means, partition around medoids, and fuzzy C‐means. These techniques have demonstrated robust performance in stratifying patient populations, identifying latent phenotypes, and improving risk classification in conditions such as HF and hypertension.^14^, ^40^, ^41^, ^42^, ^43^, ^44^, ^45^, ^46^, ^47^, ^48^, ^49^, ^50^, ^51^, ^52^, ^53^, ^54^ Evaluating cluster quality in such settings typically relies on internal validation metrics such as the Silhouette Score, Calinski–Harabasz Index, and Davies–Bouldin Index, or comprehensive packages like NbClust, which integrate multiple criteria for determining the optimal number of clusters.

For practical implementation, *Table* *3* synthesizes the key strengths and drawbacks of commonly used clustering algorithms, highlighting considerations of data structure, model assumptions, and computational efficiency.

**Table 3:** Advantages and limitation of the clustering algorithms

Clustering method	Advantages	Potential limitations
K‐means	Fast, simple to implement; performs well on large datasets	Requires predefined number of clusters (k); assumes spherical clusters; uses Euclidean distance, which is not suitable for non‐continuous or mixed‐type data
Hierarchical agglomerative clustering	No need to predefine number of clusters; produces dendrograms for visualization	Computationally expensive for large datasets; sensitive to noise
Partition around medoids	More robust to outliers than k‐means; interpretable cluster centers	Slower on large datasets; requires predefined number of clusters
Fuzzy C‐means	Allows patients to belong to multiple clusters; captures uncertainty	Difficult to interpret; requires careful tuning of parameters
Latent class models (LCM)	Probabilistic framework; handles missing data; model‐based inference	Assumes distributional form; model selection can be complex
Kamila algorithm	Specifically designed for categorical + continuous data; scalable	Less widely used; interpretability may be challenging for non‐technical users

In contrast, heterogeneous datasets, which combine variable types (e.g. demographics, biomarkers, imaging features, and clinical classifications), require more nuanced algorithmic strategies. Here, models like the latent class model (LCM) and the Kamila algorithm are particularly advantageous. The LCM assumes a probabilistic structure suited for discrete and continuous variables, while Kamila is a hybrid distance‐based method specifically designed for mixed data types.^15^, ^36^, ^55^, ^56^ These approaches not only handle variable heterogeneity more effectively but also enhance subgroup discovery, yielding clinically interpretable clusters.

Determining the number of clusters in heterogeneous data introduces additional complexity. Unlike traditional clustering, where the number of clusters may be inferred from compactness or separation metrics, model‐based approaches like LCM use statistical information criteria such as the Bayesian information criterion and integrated completed likelihood to balance model fit with complexity. Meanwhile, prediction strength—used in evaluating Kamila outputs—quantifies cluster stability across bootstrap samples, offering a more rigorous assessment of cluster reproducibility.^57^

## Internal validation of clusters

### Outcomes

After clustering the data, the next critical step is to validate the association between the identified clusters and clinically meaningful outcomes.^58^ This process verifies whether the clusters have clinical relevance and can serve as reliable predictors of patient outcomes. Common statistical methods employed include logistic regression analysis (for binary outcomes) and Cox proportional‐hazards model (for time‐to‐event data). These analyses quantify the statistical significance of the relationship between cluster membership and clinical outcomes, establishing whether the clusters are robust predictors (thus anchoring their relevance).

### Building a tool/model that can predict cluster membership

Developing a robust predictive model that assigns individual patients to the appropriate clusters is crucial for translating clustering results into usable clinical tools. *Table* *4* summarizes the major steps—algorithm selection, model training and evaluation, and performance metrics—and highlights how each step contributes to a reliable, interpretable, and scalable predictive pipeline.^14^, ^56^, ^59^, ^60^, ^61^, ^62^, ^63^ Together, these components ensure that the final model is both methodologically sound and clinically meaningful.

**Table 4:** Overview of key steps in building and validating a predictive model for cluster membership

Step	Description	Key points
Algorithm selection	Choose an appropriate classification method (e.g. decision trees, random forests, SVMs) to map patient features to cluster labels.	**Decision trees** offer transparency and ease of interpretation, making them appealing for routine clinical use.^14^, ^56^ **Random forests** combine multiple decision trees to improve accuracy and reduce overfitting, particularly useful for complex datasets.^14^, ^59^ **Support vector machines (SVMs)** capture non‐linear relationships and can perform well with high‐dimensional data.^60^, ^61^, ^62^, ^63^
Model training & evaluation	Split data into training and test sets Train using cross‐validation for robust and unbiased performance estimates	**Cross‐validation (e.g. 10‐fold)**: maximizes use of the dataset, reduces performance variance, and mitigates overfitting. **Hyperparameter tuning**: use grid/random search to optimize parameters (e.g. number of trees in random forests, kernel type in SVM) to improve predictive power. **Data preprocessing**: ensure consistent handling of missing values and outliers.
Performance metrics	Assess accuracy, interpretability, and clinical value of the model.	**Accuracy**: overall rate of correct predictions, but can be misleading with imbalanced data. **Precision & recall (sensitivity)**: especially useful when one class/cluster is rarer (high‐risk phenotypes). **Specificity**: ensures the model does not overclassify patients into a risk cluster. **F1‐score**: harmonic mean of precision and recall, balancing both. **ROC‐AUC/C‐index:** when no censored data exists, the ROC‐AUC is equivalent to the C‐index, assessing the model's discriminative ability.

## External validation of clusters

External validation is a pivotal phase that confirms the generalizability of the clustering framework and the predictive model beyond the derivation dataset. This phase includes several key components:

### Data collection for external validation

Data must be collected from independent, external sources, such as hospitals, clinics, or healthcare systems distinct from the original dataset. This diversity ensures that the model is tested on a range of patient populations, thereby enhancing its generalizability.

### Preprocessing and harmonization

External datasets may differ in format, variable definitions, and quality. Preprocessing steps are required to standardize data formats, manage missing values, and ensure consistency in variable definitions, making the external data compatible with the original clustering and prediction models.

### Assessment of model generalization

Once the external data have been preprocessed, the predictive model is applied to assign cluster membership, followed by an evaluation of the relationship between the predicted clusters and clinical outcomes to validate that the associations observed in the derivation dataset hold externally.

### Validation of the machine learning tool's clinical risk‐stratifying utility

Beyond merely predicting outcomes, the tool must be shown to enhance clinical risk prediction. For instance, a ML tool that identifies subgroups at varying stroke risk in atrial fibrillation must demonstrate an improvement in risk prediction beyond conventional scores. The combination of the C‐index (assessing discrimination) and NRI (evaluating net reclassification improvement) provides a robust assessment of the tool's practical significance.^64^, ^65^

As highlighted previously, clustering's value lies not only in risk stratification but also in guiding personalized therapeutics. Therefore, external validation should confirm whether cluster‐based differences in treatment response (e.g. placebo vs. intervention) remain consistent in independent populations. For instance, if certain clusters in the original cohort demonstrated a distinct benefit from a specific therapy, the same pattern should be observable in the external dataset to substantiate the reproducibility and clinical importance of these findings. This step is crucial to ensure that clusters retain their practical relevance and continue to inform targeted treatment decisions in diverse patient settings.

If the tool fails to improve risk stratification or predict treatment response, its clinical utility must be reconsidered. However, even if it primarily identifies patients with distinct biological profiles without immediate clinical application, it can still contribute valuable insights into disease mechanisms.

## Final step: clinical implementation

The ultimate goal of developing these predictive models is to ensure their accessibility and utility for clinical practitioners. Two distinct methodologies are proposed to facilitate clinical implementation.

### Decision tree approach

A user‐friendly graphical interface can be developed for simpler models, such as decision trees. This interface would allow physicians to input patient data and easily navigate the decision‐making process to assign patients to the appropriate clusters. Such a tool offers transparency and interpretability, akin to the decision support tools described in previous studies.^14^, ^56^

### Software for complex models

A specialized software solution is recommended for more complex predictive models (e.g. random forests, support vector machines). This software would integrate directly into clinical workflows by accepting patient data as input and providing real‐time predictions regarding cluster membership. The approach aligns with existing methodologies for risk score calculation and enhances the practical application of complex predictive models in clinical settings.^66^, ^67^

## Limitations and disadvantages of machine learning algorithms

While ML approaches offer significant advantages—such as modelling non‐linear associations and processing large, complex datasets—they are not without limitations.

### Data dependence and quality issues

Machine learning‐based clustering approaches are highly dependent on the quality, quantity, and width of available data. Inadequate or biased datasets can lead to overfitting, where models perform well in derivation cohorts but fail to generalize to external populations.^68^, ^69^ This issue is particularly pronounced in clinical research, where heterogeneity, missingness, and variability in data collection are frequent.^8^, ^70^, ^71^

Equally important, the validity and informativeness of clusters are strongly shaped by which data domains are included. As summarized in *Table* *5*, cardiovascular clustering studies could leverage up to at least 10 major domains, ranging from patient history and physical examination to imaging, omics, and digital data. Yet, most published studies have relied on only a limited subset, often restricted to patient history, clinical variables, routine lab data, and sometimes echocardiography. Consequently, clusters may be valid within this narrow framework but fail to capture broader biological or mechanistic heterogeneity.

**Table 5:** Data types commonly used in cardiovascular clustering studies

Domain	Examples
Patient history	Demographics (age, sex, BMI, ethnicity), comorbidities (diabetes, hypertension, CKD, COPD, obesity, AF), socioeconomic status, lifestyle factors (smoking, diet, physical activity), family history
Physical examination	Blood pressure, HR, oxygen saturation, oedema, jugular venous pressure, NYHA class, 6‐min walk test
Trajectory of care	Timing and number of hospitalizations, ICU stay, outpatient visit frequency, longitudinal follow‐up
Treatment data	Medications (ACEi/ARB, ARNI, beta‐blockers, MRA, SGLT2i, diuretics), devices (ICD, CRT, LVAD), revascularization, valve interventions
Laboratory/biomarkers	NT‐proBNP, troponins, creatinine/eGFR, haemoglobin, electrolytes, lipid profile, CRP, uric acid
Omics	Genomics (GWAS, polygenic scores), transcriptomics, proteomics, metabolomics, epigenetics
Conventional imaging	Echocardiography (LVEF, LV volumes, diastolic function, valvular disease), cardiac MRI (fibrosis, T1/T2 mapping, ECV), CT (calcium score, plaque burden), PET (perfusion, inflammation)
Advanced imaging/radiomics	ML‐based feature extraction from echo, CT, MRI, PET; texture analysis; automated segmentation
ECG/Holter data	Rhythm disturbances, conduction disorders, QRS duration, HR variability, AF burden, arrhythmia episodes
Digital/wearable data	Remote monitoring (BP, weight, oximetry), continuous HR, activity, sleep metrics, telehealth data

ACEi, angiotensin‐converting enzyme inhibitor; AF, atrial fibrillation; ARB, angiotensin receptor blocker; ARNI, angiotensin receptor–neprilysin inhibitor; BMI, body mass index; BP, blood pressure; CKD, chronic kidney disease; COPD, chronic obstructive pulmonary disease; CRP, C‐reactive protein; CRT, cardiac resynchronization therapy; CT, computed tomography; ECG, electrocardiogram; ECV, extracellular volume; eGFR, estimated glomerular filtration rate; GWAS, genome‐wide association study; HR, heart rate; ICD, implantable cardioverter‐defibrillator; ICU, intensive care unit; LVAD, left ventricular assist device; LV, left ventricular; LVEF, left ventricular ejection fraction; ML, machine learning; MRA, mineralocorticoid receptor antagonist; MRI, magnetic resonance imaging; NT‐proBNP, N‐terminal pro‐B‐type natriuretic peptide; NYHA, New York Heart Association; PET, positron emission tomography; SGLT2i, sodium–glucose co‐transporter 2 inhibitor.

Future work should therefore explicitly report which domains were used and critically assess how missing dimensions influence results. For instance, clusters derived from clinical and imaging data may inform prognosis but will provide limited mechanistic insights, whereas omics‐driven clusters may suggest therapeutic targets but lack immediate clinical applicability.

Concrete measures to address these challenges include systematic reporting of data coverage, validation across cohorts with varying levels of granularity, and progressive integration of multimodal datasets. Ultimately, the completeness of data dimensions must be recognized as a key determinant of the robustness, interpretability, and clinical utility of clustering in cardiovascular medicine.

### Interpretability and transparency

One of the most frequently cited concerns in the clinical use of ML is the ‘black‐box’ problem.^72^, ^73^, ^74^ Unlike traditional statistical models—such as linear regression or Cox proportional hazards models—complex ML techniques often do not provide transparent explanations for how inputs are transformed into outputs. This issue is particularly problematic in unsupervised learning, where there is no predefined outcome variable to guide or evaluate interpretability, and where cluster formation is based solely on data structure and distance metrics.

Most clustering algorithms (e.g. k‐means, hierarchical clustering, LCM) identify subgroups by optimizing mathematical criteria such as within‐cluster variance or likelihood functions. However, the resulting clusters are frequently difficult to interpret clinically. For example, a patient may be assigned to a cluster without a clear rationale or explanation as to which features drove that assignment or what clinical implications it entails. This lack of transparency can undermine clinician trust and hinder clinical decision‐making, especially when the clusters suggest therapeutic actions or prognostic risk levels.

Efforts to address these concerns are ongoing. Post hoc explainability tools such as SHapley Additive exPlanations (SHAP), and feature importance analyses can help illuminate which variables contribute most to cluster differentiation. However, these tools are still evolving, are rarely applied in unsupervised settings, and may produce inconsistent or unstable explanations. Moreover, few studies using clustering in cardiovascular research have systematically employed these techniques, and none have standardized their use for routine clinical validation.^8^, ^70^, ^75^, ^76^, ^77^

### Generalizability and external validation

A major limitation of current ML applications in cardiovascular research is the widespread absence of rigorous external validation. Most studies are conducted within a single cohort or institutional dataset, with limited testing on independent populations. As a result, the generalizability of the identified clusters—i.e. their ability to reliably classify patients outside the original derivation setting—remains uncertain. This lack of external validation has several consequences.

First, it raises the risk that the discovered clusters are data‐dependent artifacts rather than robust clinical subgroups. Clusters derived in one setting may reflect local demographic, socioeconomic, or practice‐specific characteristics that are not representative of broader populations.

Second, many clustering algorithms are sensitive to differences in data preprocessing, variable selection, and missing data handling. If external datasets are not harmonized with the derivation cohort—e.g. due to differing definitions of comorbidities or measurements of biomarkers—the application of previously developed models becomes unreliable. Inconsistent data formats and variable availability across EHR systems further exacerbate this issue.

Third, even when external validation is attempted, it is often limited to visual or descriptive comparison of cluster characteristics, rather than formal statistical validation. Without applying the original predictive model to assign new cluster membership and demonstrating preserved associations with clinical outcomes, conclusions about generalizability remain speculative.

Many ML models are trained on data from a single institution or specific cohort, and without rigorous external validation, their clinical utility remains uncertain. Continuous updates and validation are necessary to ensure that models remain relevant as patient demographics and clinical practices evolve.^70^

### Integration with clinical workflows and electronic health record systems

A critical but often overlooked barrier to implementation is the lack of integration between ML tools and real‐world clinical systems—particularly EHRs. In the United States, many hospitals use EPIC, while others rely on diverse commercial EHR platforms that vary significantly in architecture, interoperability, and data accessibility. These differences create substantial technical and regulatory hurdles for embedding ML models into clinical workflows. The absence of standardized APIs, lack of support for ML‐based clinical decision tools, and commercial interests among EHR vendors can hinder scalable deployment of unsupervised clustering solutions.

Furthermore, even when integration is technically feasible, the operational complexity of deploying a clustering‐based decision support tool—particularly one involving dynamic inputs and longitudinal updates—can slow or prevent clinical adoption. Until clustering outputs can be readily interpreted and visualized within commonly used clinical software, their real‐time utility will remain limited.

### Need for randomized controlled trials prior to clinical use

Despite the promising findings arising from observational cohorts and post‐hoc analyses of randomized trials, ML‐driven stratification tools should not be used to guide patient care in the absence of prospective randomized validation. The identification of differential treatment responsiveness across clusters in retrospective analyses represents only the hypothesis‐generating stage of precision medicine (even if performed on randomized trials). These hypotheses must be prospectively tested in adequately powered RCTs with clinical outcomes as primary endpoints. Two complementary trial designs can be envisioned: (i) using models predicting cluster membership to identify patients within clusters expected to derive greater benefit, who would then be randomized to intervention versus control/placebo; or (ii) randomizing all patients upfront to either cluster‐guided therapy (with treatment allocation informed by cluster membership) versus standard care without cluster identification. These approaches address distinct but synergistic questions. Importantly, past experiences—such as trials evaluating objective risk scores like GRACE,^78^, ^79^ which failed to improve outcomes despite robust validation—highlight the need for caution. Ultimately, well‐designed RCTs are indispensable before unsupervised clustering can be ethically and effectively implemented in clinical practice. As of September 2025, there were no ongoing randomized trials in cardiology that were prospectively testing cluster‐guided care pathways.

### Potential for bias

Machine learning models can inadvertently perpetuate or exacerbate biases present in the training data. This risk can lead to less accurate predictions for underrepresented or diverse patient groups. Mitigating these risks requires careful model design, rigorous testing, and transparent reporting.^70^

Addressing these limitations through rigorous design, validation, and refinement is essential to safely and effectively integrate ML models into clinical practice.

## Conclusion

Clustering holds great potential to advance CVD research, but its impact depends on proper implementation. To address common pitfalls, we propose a comprehensive framework combining sophisticated clustering techniques with rigorous validation, ensuring that findings are clinically meaningful and actionable. The proposed workflow (*Figure* *1*) operationalizes these steps to ensure clinically actionable outputs.

This framework provides a structured methodological guide and practical tools—from intuitive decision tree interfaces to advanced software solutions—for seamless integration into clinical practice. Enhancing risk stratification, treatment personalization, and patient outcomes bridges the gap between theoretical insights and real‐world application.

To facilitate adoption, an R‐based implementation of this framework is available at https://gitlab.com/cic‐p/cv‐clustering. This enables researchers and clinicians to apply these advanced clustering methods effectively in CVD research.
